# Monocyte Chemotactic Protein-1 Promotes the Myocardial Homing of Mesenchymal Stem Cells in Dilated Cardiomyopathy

**DOI:** 10.3390/ijms14048164

**Published:** 2013-04-15

**Authors:** Jing Guo, Haifeng Zhang, Junjie Xiao, Jian Wu, Yong Ye, Zheng Li, Yunzeng Zou, Xinli Li

**Affiliations:** 1 Department of Cardiology, First Affiliated Hospital, Nanjing Medical University, Nanjing 210029, China; E-Mails: gugudan@gmail.com (J.G.); haifeng_zhang@163.com (H.Z.); junjiexiao@live.cn (J.X.); 2 Cell metabolism lab, School of Life Science, Shanghai University, Shanghai 200444, China; 3 Shanghai Cardiovascular Disease Institute, Fudan University, Shanghai 200032, China; E-Mails: everwoo@sina.com (J.W.); yeyongleo@gmail.com (Y.Y.); leezheng1985@hotmail.com (Z.L.)

**Keywords:** monocyte chemotactic protein-1, mesenchymal stem cells, dilated cardiomyopathy, myocardial, homing

## Abstract

Dilated cardiomyopathy (DCM) is the most common form of non-ischemic cardiomyopathy that leads to heart failure. Mesenchymal stem cells (MSCs) are under active investigation currently as a potential therapy for DCM. However, little information is available about the therapeutic potential of intravenous administration of MSCs for DCM. Moreover, how MSCs home to the myocardium in DCM is also unclear. DCM was induced by intraperitoneally administering Doxorubicin and MSCs or vehicles were infused through the internal jugular vein. Cardiac functions including the percentage of fractional shortening, left ventricular diastolic dimension, left ventricular end-diastolic pressure, and left ventricular maximum d*p*/d*t* were evaluated by echocardiographic and hemodynamic studies. Fibrosis was determined by Masson’s trichrome staining. The mRNA expression levels of monocyte chemotactic protein-1 (MCP-1), stromal cell-derived factor-1 (SDF-1), macrophage inflammatory protein-1α (MIP-1α), and monocyte chemotactic protein-3 (MCP-3) were determined using real time polymerase chain reactions and the protein expression level of MCP-1 was detected with Western blot. The MSCs expression of C-C chemokine receptor type 2 (CCR2), a MCP-1 receptor, was confirmed by Western blot and flow cytometry analysis. The chemotactic effects of MCP-1/CCR2 were checked by assessing the migration *in vitro* and *in vivo*. MSCs transplantation improved the cardiac function and decreased the myocardial fibrosis of mice with DCM. MCP-1 was up-regulated in dilated myocardial tissue both at the mRNA and protein level while SDF-1, MIP-1α and MCP-3 remain unchanged. CCR2 was present in MSCs. MCP-1 promoted MSCs migration *in vitro* while CCR2 inhibition decreased the migration of MCP-1 to the dilated heart. This study provides direct evidences that peripheral intravenous infusion of MSCs can support the functional recovery of DCM. In addition, novel insights into the myocardial homing factor of MSCs in DCM are presented. Modulation of MCP-1/CCR2 signaling system might be a novel therapeutic strategy for DCM.

## 1. Introduction

Dilated cardiomyopathy (DCM) is the most common form of non-ischemic cardiomyopathy leading to heart failure [1,2]. DCM accounts for approximately 10% of patients with heart failure [3]. Heart failure is associated with high morbidity and mortality [1,4]. Currently heart transplantation is the only effectively therapy for DCM at the end stage [5]. However, due to the strict selection criteria and chronic shortage of donor hearts, most patients do not have the chance to receive a transplant [5]. Therefore, preventing the progression of myocardial dysfunction in DCM is a major challenge requiring novel therapeutic strategies [2,4].

Mesenchymal stem cells (MSCs) have a powerful proliferative potential and possess the ability of differentiating into various cell lineages [6–8]. In fact, MSCs are under active investigation as a potential therapy for diverse cardiovascular diseases with the hope of restoring dysfunctional heart [6]. *In vitro*, after 5-azacytidine treatment, MSCs are able to differentiate into beating cardiomyocytes [9]. *In vivo*, after being directly injected into an infracted heart, MSCs can help maintain the function of the broken heart [10]. Several studies have pointed out that directly injection of MSCs into the myocardium of DCM could induce myocardial regeneration and improve cardiac function both in animals and human [11–13]. Interestingly, a pilot study of intracoronary bone marrow MSCs infusion in DCM patients has proved a significant improvement in the left ventricular ejection fraction (LVEF) and New York Heart Association (NYHA) Functional Classification [14]. Moreover, the TOPCARE-DCM study showed that intracoronary administration of bone marrow MSCs was associated with regional and global improvement in the LVEF [15]. However, little information is available about the therapeutic potential of intravenous administration of MSCs for DCM [1].

According to the results from most reports in the literature regarding the therapeutic value of MSCs in DCM, only modest effects on cardiac function were observed [1]. Thus, a significant improvement of MSCs-based therapy is highly required before widespread clinical use. One potential approach would be to increase homing of MSCs to the myocardium [1,11]. However, currently little is known about how MSCs home to the myocardium in DCM [11,16]. Monocyte chemotactic protein-1 (MCP-1), stromal cell-derived factor-1 (SDF-1), macrophage inflammatory protein-1α (MIP-1α), and monocyte chemotactic protein-3 (MCP-3) are four stem cell chemo-attractant cytokines that are thought to play a role in post-infarct cardiac repair. Nevertheless, it is unclear whether any of the above cytokines responsible for the myocardial homing of MSCs in DCM. Thus, this study aims at determining the therapeutic effects of intravenously administered MSCs in DCM and also identifying a candidate factor that mediates the myocardial homing of MSCs in DCM.

## 2. Methods

All animal experiment protocols in this study were approved by the Animal Care and Use Committee of Nanjing Medical University. The investigation conforms to the Guide for the Care and Use of Laboratory Animals published by the US National Institutes of Health (NIH Publication No. 85-23, revised 1996).

### 2.1. Generation of Doxorubicin-Induced DCM

Doxorubicin-induced DCM was generated as previously described [5,17]. Briefly, 8–12 weeks C57/BL6 male mice were used for experiments involving cell transplantation. Doxorubicin (Sigma, St. Louis, MO, USA) was administered intraperitoneally with six equal injections (each containing 2.5 mg/kg) over a period of two weeks for a total dose of 15 mg/kg. For mice in control group, equal volume of physiological saline was injected. Four weeks later, ventricular function was assessed by echocardiography.

### 2.2. Expansion and Transplantation of Bone Marrow MSCs

The expansion of MSCs was performed as previously described [11]. In brief, 8–12 weeks C57/BL6 male mice were used to harvest bone marrow by flushing the femurs with DMEM/F12 (Gibco) using a 20-gauge needle. After that, bone marrow cells were cultured in DMEM/F12 (Gibco) supplemented with 10% fetal bovine serum (Gibco) and 1% antibiotics (Sigma). A small number of cells developed visible symmetric colonies by day 5 to 7. Thereafter, non-adherent hematopoietic cells were removed, and the medium was replaced. The adherent, spindle-shaped MSC population expanded to over 5 × 10^7^ cells within 4 to 5 passages after the cells were first plated.

Four weeks after final doxorubicin injection, a total of 5 × 10^7^ MSCs/100 μL phosphate-buffed saline (PBS), or PBS alone were slowly infused through the internal jugular vein. Sham mice received internal jugular vein infusion of 100 μL PBS. This protocol resulted in the creation of 3 groups as follows: DCM mice given PBS (untreated DCM group), DCM mice given MSCs (MSC-treated DCM group), and sham mice given PBS (untreated sham group).

### 2.3. Echocardiographic and Hemodynamic Studies

Echocardiographic studies were performed before MSC treatment and also 4 weeks after cell transplantation. Two-dimensional, targeted M-mode tracings were obtained at the level of the papillary muscles with an echocardiographic system (Visual Sonics Inc., Toronto, ON, Canada). In the long-axis view, left ventricular end-systolic (LVESD) and left ventricular end-diastolic chamber diameters (LVEDD) and thickness of the interventricular spetum and the posterior wall were determined. After that, left ventricular fractional shortening [FS = (LVEDD − LVESD)/LVEDD × 100] was calculated. All measurements were averaged for at least three consecutive cardiac cycles and were carried out by two experienced technicians who were unaware of the identities of the respective experimental groups.

Hemodynamic studies were performed 4 weeks after cell transplantation. A 1.4 F pressure catheter (SPR 671, Millar Instruments) was inserted into the aorta and left ventricle through the right common carotid artery. The transducer was connected to Power Lab system (AD Instruments, Castle Hill, Australia) and left ventricular systolic and end-diastolic pressures, left ventricular maximum d*p*/d*t* (LVmd*p*/d*t*) were recorded.

### 2.4. Fibrosis Determination

Part of the left ventricular myocardium was fixed in 10% formalin, cut transversely, embedded in paraffin, and stained with Masson’s trichrome. Transverse sections were randomly obtained from the 3 levels (basal, middle and apical), and at least 5 randomly selected fields per section were analyzed. Digital photographs were taken using a high-resolution digital image analysis system (QwinV3; Leica, Wetzlar, Germany).Collagen volume fraction was calculated as the sum of all areas containing connective tissue divided by the total area of the image using Image-Pro Plus 5.0.

### 2.5. RNA Extraction and Real-Time Polymerase Chain Reactions (PCRs) Analysis

Total mRNA was extracted from part of cardiac tissues using Trizol reagent (Invitrogen, Carlsbad, CA, USA) according to the manufacturer’s instruction. Real-time PCRs with SYBR Green, which was validated with respect to reproducibility and linearity within the measuring range, was performed in quadruplicate with the Cycler System (Bio-Rad, Hercules, CA, USA), and Power SYBR Green PCR Master Mix (TaKaRa, Shiga, Japan) as reagent. To correct for potential variances between samples in mRNA extraction or in reverse transcribed efficiency, the mRNA content of each gene was normalized to the expression of the stably expressed reference gene glyceraldehyde-3-phosphate dehydrogenase (GAPDH) within the same sample. cDNA sequences were obtained from GenBank sequence database of the National Center for Biotechnology Information [18], and primers were designed with Primer3 software [19]. Sequences for all PCR primers were as follows: MCP-1, Forward, 5′-GCATCCACGTGTTGGCTCA-3′ and Reverse, 5′-CTCCAGCCTACTCATTGGGATCA-3′; SDF-1, Forward, 5′-CAGAGCCAACGTCAAGCATC-3′ and Reverse, 5′-TTAATTTCGGGTCAATGCACAC-3′; MIP-1α, Forward, 5′-CATGACACTCTGCAACCAAGTCTTC-3′ and Reverse, 5′-GAGCAAAGGCTGCTGGTTTCA-3′; MCP-3′, Forward, 5′-CAGCTCTCACTGAAGCCAGCTC-3′ and Reverse, 5′-AGCAGCATGTGGATGCATTG-3′; GAPDH, Forward, 5′-CCACTCTTCCACCTTCGATG-3′ and Reverse 5′-TCCACCACCCTGTTGCTGTA-3′. The cycling parameters were as follows: denaturation at 94 °C for 1 min; annealing at 55–60 °C for 1 min (depending on the primer); and elongation at 72 °C for 1 min (40 cycles). All real time PCR reactions, including no-template controls, were performed in triplicate. The amplification products were assessed using melting curve analysis. The relative expression ratios of mRNAs were determined using the crossing point as the cycle number. The relative expression level for each mRNA was calculated using the 2^−ΔΔ^*^C^*^t^ method.

### 2.6. Western Blot Analysis

MSCs and part of cardiac tissues were lysed in RIPA buffer (20 mM Tris, 150 mM NaCl, 1% Nonidet P-40, 0.1% sodium dodecylsulfate. and 10 μg/mL aprotinin). Fifty micrograms of protein from each sample were separated by SDS-PAGE followed by being transfered to nitrocellulose membranes. The membranes were then incubated overnight at 4 °C, with anti-MCP-1 (1:1000, Novus Biologicals, Littleton, CO, USA) or C-C Chemokine Receptor Type 2 (CCR2, 1:1000, Abcam, Cambridge, MA, USA) or GAPDH (1:1000, Abcam, Cambridge, MA, USA) antibodies. After incubation with the secondary antibody conjugated with horseradish peroxidase, membranes were extensively washed, and the detection of HRP was performed by Las3000 (Fujifilm Tokyo, Japan).

### 2.7. Flow Cytometry Analysis

After treated with 0.125% trypsin-EDTA, MSCs were harvested and washed twice with PBS. After that, MSCs were incubated with primary rabbit monoclonal antibodies anti-CCR2 (1:200, Abcam, Cambridge, MA, USA) in 1% bovine serum albumin for 1 h at 4 °C and anti-mouse immunoglobulin G (IgG) labeled with fluorescein isothiocyanate (1:200, Abcam, Cambridge, MA, USA) for 1 h. IgG isotype antibodies (Abcam, Cambridge, MA, USA) were used as control. MSCs were then washed in PBS, resuspended in 0.5 mL PBS and assayed in a flow cytometer (Beckman Coulter, Miami, FL, USA). As an isotype control, nonspecific mouse immunoglobulin (Abcam, Cambridge, MA, USA) was substituted for the primary antibody.

### 2.8. *In Vitro* Migration Assay

Migration assays were used to investigate the chemotactic effect of MCP-1 to MSCs. Briefly, 5 × 10^4^ MSCs were placed in the upper chambers of Costar 24-well transwell plates with 5-μm pore filters (Corning, New York, NY, USA). DMEM/F12 containing 1% FBS of 600 μL (GIBCO, Grand Island, NY, USA) alone or containing 20, 100, 300 and 500 ng/mL MCP-1 (R&D, Minneapolis, MN, USA) were placed in the lower chambers or wells. CCR2 antibody (E68) (Abcam, Cambridge, MA, USA) were used (10 μg/mL) in the inhibition test, and IgG isotype antibodies (Abcam, Cambridge, MA, USA) were used as control. After incubating plates for 24 h at 37 °C, migrated cells were collected from the lower chambers and counted.

### 2.9. *In Vivo* Migration Assessment

Lentiviral vectors expressing shRNAs targetting CCR2 or control vectors (pLKO.1-shGFP) were constructed as previously described [20]. Twenty-four hours after the intravenously transplantation of CCR2 shRNAs or control vectors infected MSCs, mice were sacrificed and the hearts were removed. A Kodak *in vivo* imaging system FX Pro (Kodak, Rochester, NY, USA) was used for GFP detection. The captured images were processed and analyzed with the Kodak imaging system equipped with Carestream MI software. Fluorescence mean intensity was used to represent the quantity of MSCs in the heart.

### 2.10. Statistical Analysis

The data were expressed as the mean ± SE. An independent-samples *t*-test or one-way ANOVA was conducted to evaluate the one-way layout data. If a significant difference was observed, Bonferroni’s post-hoc test was conducted to identify groups with significant differences. *p*-Values that were less than 0.05 were considered to be statistically significant.

## 3. Results

### 3.1. MSCs Transplantation Improves the Cardiac Function of DCM Mice

**Figure 1:**
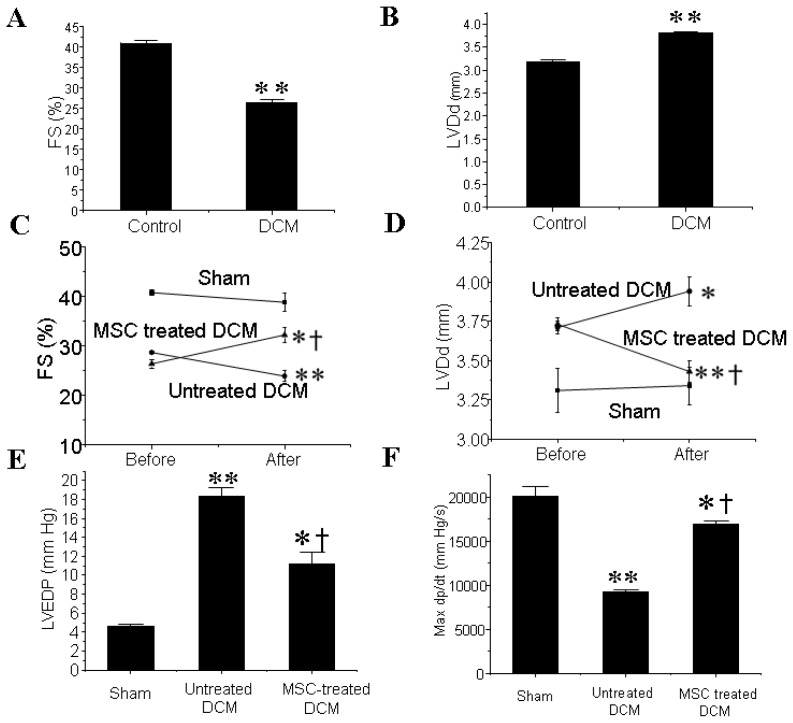
Mesenchymal stem cells (MSCs) transplantation improves cardiac function of dilated cardiomyopathy (DCM); (**A**) Percentage of left ventricular fractional shortening (FS%) is decreased in DCM. ** *p <* 0.01 *vs*. control group; *n* = 4 per group; An independent-samples *t*-test was conducted; (**B**) Left ventricle end-diastolic diameter (LVDd) is elevated in DCM. ** *p <* 0.01 *vs*. control group; *n* = 4 per group; An independent-samples *t*-test was conducted; (**C**) MSCs transplantation improves FS% of DCM. ** *p* < 0.01 *vs*. before transplantation; * *p* < 0.05 *vs*. before transplantation; ^†^*p <* 0.01 *vs*. the time-matched untreated DCM group; *n* = 5 per group; A one-way ANOVA was conducted. If a significant difference was observed, Bonferroni’s post-hoc test was conducted to identify groups with significant differences; (**D**) MSCs transplantation preserves LVDd of DCM. ** *p* < 0.01 *vs*. before transplantation; * *p* < 0.05 *vs*. before transplantation; ^†^*p <* 0.01 *vs*. the time-matched untreated DCM group; *n* = 5 per group; A one-way ANOVA was conducted. If a significant difference was observed, Bonferroni’s post-hoc test was conducted to identify groups with significant differences; (**E**) MSCs transplantation preserves left ventricle end-diastolic pressure of DCM. ** *p <* 0.01 *vs*. sham group; * *p <* 0.05 *vs*. sham group; ^†^*p <* 0.05 *vs.* the time-matched untreated DCM group. *n* = 5 per group; A one-way ANOVA was conducted. If a significant difference was observed, Bonferroni’s post-hoc test was conducted to identify groups with significant differences; (**F**) MSCs transplantation improves left ventricle maximum d*p*/d*t*. ** *p <* 0.01 *vs*. sham group; * *p <* 0.05 *vs.* sham group; ^†^*p <* 0.05 *vs*. the time-matched untreated DCM group. *n* = 5 per group; A one-way ANOVA was conducted. If a significant difference was observed, Bonferroni’s post-hoc test was conducted to identify groups with significant differences.

Four weeks after the final injection of doxorubicin, a significant decrease in the percentage of FS (FS%) (Figure 1A) and a marked dilation of left ventricle (Figure 1B) were observed in the DCM group compared with the control group as determined by echocardiography. MSCs transplantation significantly increased FS% and also inhibited the augment of left ventricular diastolic dimension (LVDd) in DCM mice (Figure 1C,D).

Hemodynamic study further revealed that the elevation of left ventricular end-diastolic pressure (LVEDP) in DCM group was significantly attenuated after MSCs engraftment. In addition, LVmd*p*/d*t* was significantly higher in the MSCs group than in the untreated DCM group (Figure 1E,F).

### 3.2. MSCs Transplantation Reduces Myocardial fibrosis

**Figure 2:**
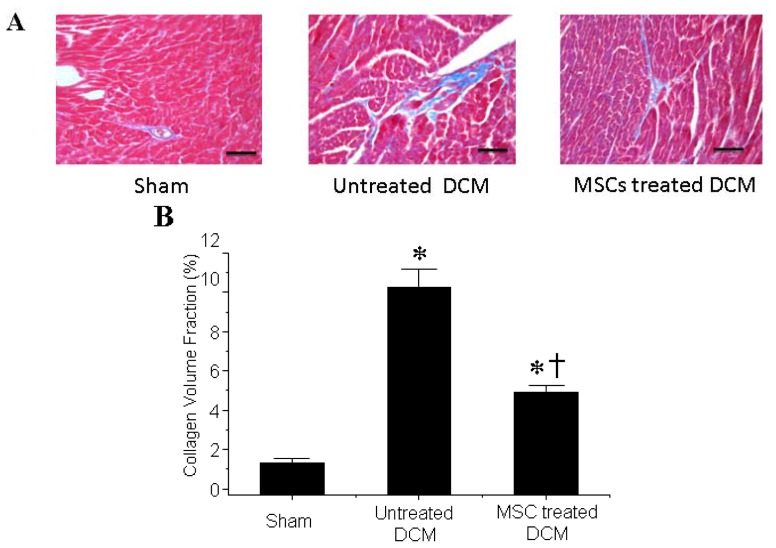
Mesenchymal stem cells (MSCs) transplantation reduces myocardial fibrosis of dilated cardiomyopathy (DCM); (**A**) Representative myocardial sections stained with Masson’s trichrome. Scale bars = 40 μm; (**B**) MSCs transplantation decreases collagen volume fraction of DCM. * *p* < 0.01 *vs.* sham group; ^†^*p* < 0.01 *vs*. untreated DCM group. *n* = 5 per group; A one-way ANOVA was conducted. If a significant difference was observed, Bonferroni’s post-hoc test was conducted to identify groups with significant differences.

Cardiac fibrosis was attenuated in MSCs-treated DCM group comparing to the untreated DCM group as determined by Masson’s trichrome staining (Figure 2A). Quantitative analysis further demonstrated that the collagen volume fraction in the MSCs-treated DCM group was significantly smaller than that in the untreated DCM group (Figure 2B).

### 3.3. MCP-1 Is Up-regulated in Dilated Myocardial Tissue

**Figure 3:**
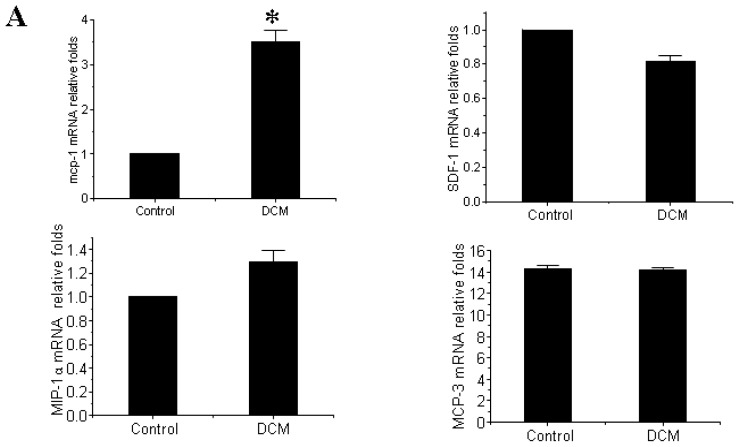

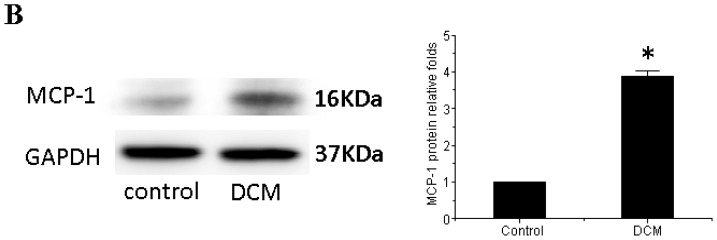
Monocyte Chemotactic Protein-1 (MCP-1) is a candidate myocardial homing factor of mesenchymal stem cells in dilated cardiomyopathy (DCM); (**A**) The mRNA expression level of MCP-1 is significantly up-regulated in DCM compared with control while SDF-1, MIP-1α and MCP-3 remain unchanged. * *p* < 0.01 *vs.* control group; (**B**) MCP-1 is increased in DCM comparing to the control at the protein level. * *p* < 0.01 *vs*. control group. *n* = 3 per group; An independent-samples *t*-test was conducted.

The mRNA expression level of MCP-1, SDF-1, MIP-1α and MCP-3, four previously reported important chemokines in the myocardial infarction, were firstly detected in this study to indicate if anyone might contribute to the myocardial homing of MSCs in DCM [6,17,21,22]. As shown in Figure 3A, the mRNA expression level of MCP-1 was significantly up-regulated in DCM compared with control while SDF-1 (*p* = 0.507), MIP-1α (*p* = 0.728) and MCP-3 (*p* = 0.784) remain unchanged. Western blot further confirmed that the increase of MCP-1 in DCM comparing to the control at the protein level (Figure 3B).

### 3.4. CCR2, a MCP-1 Receptor, Is Present in MSCs

**Figure 4:**
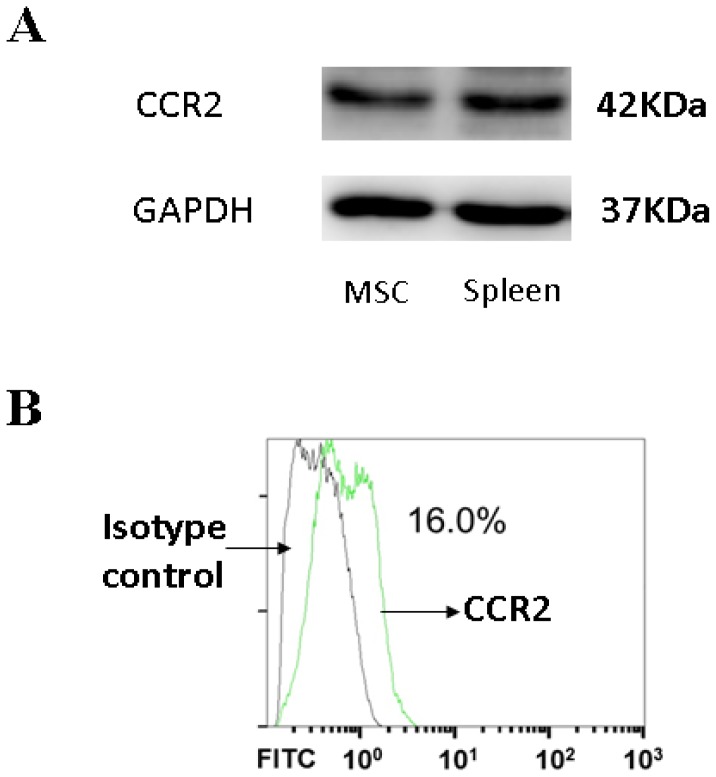
C-C chemokine receptor type 2 (CCR2) is present in mesenchymal stem cells (MSCs); (**A**) CCR2 is present in MSCs as indicated by Western blot; (**B**) The presence of CCR2 in MSCs is confirmed by flow cytometry analysis.

CCR2 is a cognate receptor of MCP-1 [23]. The binding of MCP-1 to CCR2 is a presupposition for the mediation of the homing of MSCs [23]. CCR2 was present in MSCs as shown by Western blot (Figure 4A). Moreover, the presence of CCR2 on the cell membrane was also confirmed by flow cytometry analysis (Figure 4B).

### 3.5. MCP-1 Promotes MSCs Migration *in Vitro*

**Figure 5:**
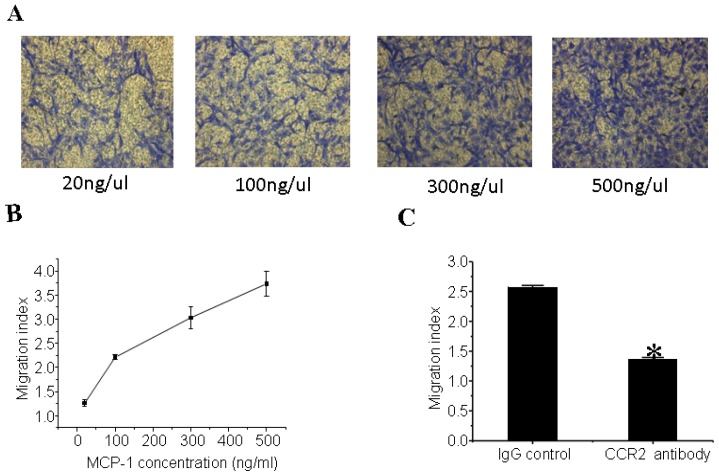
Monocyte Chemotactic Protein-1 (MCP-1) causes mesenchymal stem cells (MSCs) chemotaxis *in vitro*; (**A**) Representative photographs of migrated MSCs staining with crystal violet. The migration of MSCs is dose-dependently increased upon MCP-1 treatment; (**B**) MCP-1 treatment dose-dependently promotes MSCs migration; (**C**) C-C chemokine receptor type 2 antibody pretreatment abolishes the effects of MCP-1 treatment. * *p* < 0.01, compared with IgG control group. An independent-samples *t*-test was conducted.

The representative photographs of migrated MSCs staining with crystal violet were shown in Figure 5A. As indicated in Figure 5B, the migration of MSCs was dose-dependently increased upon MCP-1 treatment. After pretreatment with the CCR2 antibody, MSCs failed to migrate compared to pretreatment with the isotype control antibody (Figure 5C), indicating that CCR2 is required for the effects of MCP-1 in promoting MSCs migration.

### 3.6. CCR2 Inhibition Decreases MSCs Migration to the Dilated Heart

**Figure 6:**
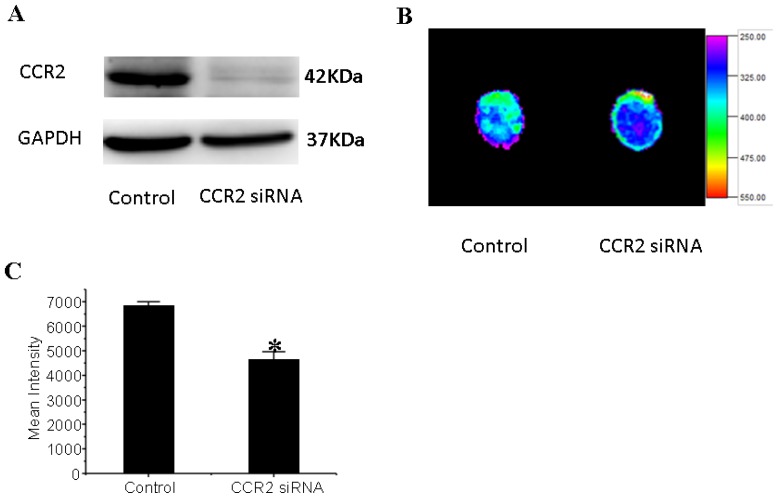
C-C chemokine receptor type 2 (CCR2) inhibition decreases mesenchymal stem cells (MSCs) migration to the dilated heart; (**A**) CCR2 siRNA decreases the expression of CCR2 in MSCs (**B**) Representative photographs of Fluorescence imaging of the migrated GFP-labled control MSCs or CCR2 siRNA MSCs to the dilated heart (**C**) CCR2 inhibition decreases MSCs migration to the dilated heart. * *p* < 0.01 *vs*. control. *n* = 3 per group; An independent-samples *t*-test was conducted.

MSCs were transduced with a CCR2 shRNA construct by lentivirus-mediated gene transfer. A significant CCR2 suppression was confirmed by Western Blot (Figure 6A). To determine whether MCP-1/CCR2 signaling plays an essential role in MSCs myocardial homing in DCM, control MSCs or CCR2 knock-down MSCs were injected equally into the mice with DCM via jugular vein. Using Fluorescence imaging to quantity the MSCs that engraftment into the heart, we found that CCR2 inhibition significantly decreased the amount of MSCs that migrated to the myocardial in DCM (Figure 6B,C), indicating that MCP-1/CCR2 signaling is required for MSCs homing in DCM.

## 4. Discussion

The treatment of DCM is always a puzzle to the physicians all over the world, and cell therapy brings a glimmer of hope [1]. Embryonic stem cells, foetal cardiomyocytes, human umbilical cord-derived cells, resident cardiac stem cells, adipose-derived stem cells, skeletal myoblasts, MSCs, and endothelial progenitor cells have been widely explored for cardiac repair [1,24,25]. Among them, MSCs may be an optimal cell type in regenerative therapy with consideration of no ethic problems and easy acquirement [17]. The therapeutic potential of MSCs for myocardial infarction and ischemic heart disease has been widely explored [1,10,26]. However, little information is available regarding its therapeutic value in DCM [1]. To the best of our knowledge, this study firstly reports the therapeutic effect of peripheral intravenous infusion of MSCs on DCM in mice, which include the improvement of cardiac function and the decrease of myocardial fibrosis.

Stem cell therapy relies on the capacity of stem cells homing to and engrafting into the appropriate target tissue [1]. But so far the homing of stem cells into heart is with extremely poor efficiency, raising an issue that how homing process can be promoted [16]. Therefore, elucidation of mechanisms guiding the homing of transplanted stem cell is important [1,6,16]. MCP-1, SDF-1, MIP-1α and MCP-3 are the most widely reported MSC homing factors in acute myocardial infarction [6,17,21,22]. However, relatively little MSCs homing factors have been indicated in DCM [1,16,17]. In the present study, we firstly found that MCP-1 was up-regulated in the dilated myocardial tissue both at the mRNA and protein level while the mRNA level of SDF-1, MIP-1α and MCP-3 remain unchanged. Moreover, similar to another report [27], CCR2, a MCP-1 receptor, was identified in MSCs. Thus, further attention is focused on MCP-1 in the present study. We found that MCP-1 promoted MSCs migration *in vitro* while CCR2 inhibition decreased MSCs migration to the dilated heart, which is consistent with the previous report [28]. Taken together, these data firstly establishes that MCP-1 is a myocardial homing factor of MSCs in DCM.

MCP-1 is a member of the C-C motif chemokine ligand-2 (CCL2) chemokines family of proteins that has been reported to induce leukocyte migration to the inflammatory tissues and organs [29,30]. Moreover, MCP-1 can also be secreted by primary breast tumors and thereafter stimulate the migration of MSCs to tumor lesions [28]. Besides that, MCP-1 also exerts nonchemotaxic effects including the induction of adhesion molecules expression, tissue factor secretion, and smooth muscle cell proliferation [23,29]. MCP-1 has also been implicated in numerous steps along the way to post-infarction heart failure: in the development of atherosclerosis, in atherosclerotic plaque instability, in recruitment of monocytes to the heart following myocardial infarction, and in post-infarction left ventricular remodeling [30–33]. The action of MCP-1 is mediated by the binding of MCP-1 to its receptor, CCR2 [34–36]. Although MCP-1 is a myocardial homing factor of MSCs in DCM as presented in this study, additional factors might contribute to the homing, which is supported by the fact that CCR2 inhibition significantly decreased but not totally prevented the migration of MSCs to the myocardial in DCM. However, this study for the first time provides direct evidences that MCP-1/CCR2 axis is at least partly responsible for the myocardial homing factor of MSCs in DCM and also indicates that new therapeutic options providing MCP-1 or CCR2 to the myocardium may become necessary for the treatment of DCM patients. Providing a conductive environment for efficient homing of endogenous MSCs may therefore become a promising novel therapeutic option for patients suffering from DCM.

Several potential limitations of this study should be highlighted. Firstly, the exact mechanism by which MSCs improve heart function is unclear. Further studies need to be performed to determine whether MSCs differentiate into cardiomyocytes, cause transdifferentiation, or have a paracine effect. It is also important to demonstrate the characteristic of CCR2 shRNA-transduced MSCs, which is the ability to differentiate into cardiomyocytes and to secret various cytokines, and the survival under ischemic condition. Alternatively, detailed assessment of cardiac function and fibrosis following injection of the CCR-2 knock-down stem cells should be conducted in the future. Secondly, although improved cardiac function was demonstrated following administration of MSCs, a direct relationship between the two was not shown. MSC trapping in the lungs may be sufficient to render myocardial benefit in the absence of MSC engraftment in the heart. Therefore, it would be valuable to demonstrate whether engrafted MSCs were detectable throughout the heart following necropsy of the animals with improved function. Thirdly, as ligands other than MCP-1 also bind to CCR-2 (e.g., MCP-2, MCP-4, MCP-5, HIV-tat), therefore the decrease in MSC migration to the heart may not be entirely MCP-1 mediated. In addition, the expression level of MCP-1 in other tissues is unclear in this study. Fourthly, it would be interesting to know whether intravenous infusion of MSCs would migrate into various tissues or not and to investigate the efficiency of MSCs migration into the heart. However, as the aim of this study is to explore the myocardial homing of MSCs, we will try to do it in our next study. However, as peripheral intravenous infusion of MSCs is less invasive than intracoronary infusion and directly injection of MSCs into the myocardium of DCM, it deserves to explore the novel way of enhancing the myocardial homing of MSCs. Lastly, the function improved and fibrosis decreased in the dilated cardiomyopathy heart following the MSC was present in this study, it deserves to investigate into whether any inflammation factors have influenced the outcome in the future.

Taken together, the present study provides novel direct evidences that peripheral intravenous infusion of MSCs can support the functional recovery of DCM. Moreover, this study also provides new insights into the myocardial homing factor of MSCs in DCM. Modulation of MCP-1/CCR2 signaling system may be a novel therapeutic strategy for DCM.
